# You are doubly excluded: the interplay of cyberostracism and perceived parental phubbing in predicting problematic smartphone use

**DOI:** 10.1016/j.abrep.2026.100719

**Published:** 2026-06-19

**Authors:** Mehran Mohammadi, Nikzad Ghanbari

**Affiliations:** a Department of Psychology, Faculty of Psychology and Educational Sciences, Shahid Beheshti University, Tehran, Iran

**Keywords:** Cyberostracism, Parental phubbing, Problematic smartphone use (PSU), Social exclusion, Temporal need-threat model, Rejection sensitivity model

## Abstract

Problematic smartphone use (PSU) poses increasing risks to young adults' psychological well-being and daily functioning. While offline social exclusion has been linked to maladaptive behaviors, the effects of cyberostracism—online forms of exclusion—and the mediating role of parental behaviors remain underexplored. Guided by the Need-Threat Model of Ostracism and the Rejection Sensitivity Model, the present study examined whether perceived parental phubbing mediates the relationship between distinct forms of cyberostracism and PSU among Iranian young adults. A total of 428 participants (303 females, 125 males; mean age = 28.03 years, SD = 8.67) completed validated self-report measures assessing cyberostracism (direct, indirect, and ignored), perceived parental phubbing, and PSU. Structural equation modeling was used to test direct and indirect pathways. Results indicated that cyber indirect exclusion was directly associated with higher PSU, whereas the association between direct exclusion and PSU was mediated by increased perceptions of parental phubbing. Cyber-ignoring was not significantly associated with PSU. These findings suggest that the psychological correlates of social media–based exclusion on smartphone use vary according to the type of exclusion, with perceived parental phubbing serving as a key process through which explicit online exclusion is associated with maladaptive smartphone engagement. Overall, these findings underscore the importance of integrating social media exclusion experiences with family-level digital behaviors into theoretical models and targeted interventions aimed at reducing PSU.

## Highlights

• Different forms of cyberostracism show differential associations with problematic smartphone use (PSU).
• Indirect cyberostracism predicts PSU directly, while direct cyberostracism operates via perceived parental phubbing.
• Cyber-ignoring showed no significant association with PSU.
• Perceived parental phubbing serves as an interpersonal mediator between online exclusion and PSU.

## 1 Introduction

The rapid expansion of digital technologies has transformed the way individuals interact, work, and manage daily life. Among these technologies, smartphones have become a ubiquitous tool across age groups, providing unprecedented convenience in communication, information access, and entertainment (Wang et al., 2020; Wang, Mao, et al., 2022; Yu et al., 2025). While these devices facilitate social connection, their pervasive use has raised growing concerns about problematic smartphone use (PSU) (Crowhurst & Hosseinzadeh, 2024; Geng et al., 2021). PSU refers to a compulsive and excessive pattern of smartphone penetration that is hard to control, often resulting in emotional distress, functional impairment in daily life and even potential harm to oneself or others (Busch & McCarthy, 2021; Elhai et al., 2020; Peltz et al., 2023; Pera, 2020; Zhang, Yuan, et al., 2023).

Empirical evidence links PSU to a wide range of negative repercussions, including anxiety and depression (Augner et al., 2023; Nikolic et al., 2023; Sun & Tang, 2025), low self-esteem (Jose et al., 2024; Sun & Tang, 2025), sleep disturbances (Elsheikh et al., 2024; Ong et al., 2024) and decreased physical activity (Demir et al., 2025; Le Steunf et al., 2024). In academic and occupational settings, PSU has also been associated with procrastination and productivity loss (Chen & Lyu, 2024; Kutluay & Karaca, 2024; Zhou et al., 2024) as well as numerous other detrimental outcomes. Therefore, there is a growing need to further examine which individuals are more vulnerable and why they are motivated toward maladaptive smartphone use (Busch & McCarthy, 2021), in order to design more targeted prevention and intervention strategies.

### 1.1 Cyberostracism and problematic smartphone use

With the extensive adoption of mobile devices, social networking sites have become an integral part of young adults' daily routines, allowing them to remain constantly connected and to strengthen their offline relationships (Smith et al., 2017; Tang & Duan, 2023). SNS users often seek connection and have expectations regarding the speed or frequency of responses from others (Hayes et al., 2016), in other words, likes or comments on social media have been found to serve a similar role to positive feedback and social support in face-to-face interactions (Smith et al., 2017; Zell & Moeller, 2018). Recent research shows that even subtle, seemingly insignificant digital events, such as not being tagged in photos on Instagram, can be perceived as aversive cues of social rejection, particularly among individuals with a high need to belong (Büttner & Rudert, 2022). Therefore, with the growing integration of social dynamics into digital platforms, users now face a greater likelihood that their online communications will not be accompanied by their desired feedback (Shi et al., 2025; Wang et al., 2025). Such an unpleasant experience is known as cyber-ostracism, which occurs in non–face-to-face electronic interactions (e.g., chat-room discussions) when individuals feel ignored or rejected (Schneider et al., 2017; Wang et al., 2024), particularly by socially significant others (Hayes et al., 2018). Cyber-ostracism can manifest in three forms (Hatun & Demirci, 2022): direct exclusion (e.g., being explicitly blocked or removed), indirect exclusion (e.g., being left out of posts, group messages, or live actions), and cyber-ignoring (e.g., not reading, responding to one's posts, messages, or questions in cyberspace).

Similar to offline ostracism, cyber-ostracism has been shown to produce comparable negative outcomes (Wang, Mu, et al., 2022; Xing & Kuo, 2024), including depression (Ding et al., 2022; Ding et al., 2025; Niu et al., 2018), aggressive behaviors (Xing & Kuo, 2024), social anxiety (Wang et al., 2024), and other adverse emotional and behavioral responses (Pamuk & Cırcır, 2023; Tang & Duan, 2023; Xu et al., 2023). Moreover, in line with the Temporal Need-Threat Model of ostracism (TNTM; Williams, 2009), an increasing amount of research suggests that experiences of cyber-ostracism undermine individuals' fundamental needs for belonging, self-esteem, control, and meaningful existence (Borawski, 2022; Galbava et al., 2021; Smith et al., 2017). After the initial reflexive pain response, individuals enter a reflective stage in which they appraise and attribute the meaning and importance of the ostracism episode. Depending on their assessment of the severity and nature of the exclusion (different forms of cyberostracism), they choose a coping strategy, primarily seeking reconnection to fortify threatened needs. However, when ostracism is severe and persists over time, individuals' coping resources become depleted, leading to a new stage called resignation, characterized by withdrawal and alienation, in which individuals no longer engage in active coping within the environment where the exclusion occurred (Williams, 2009).

Therefore, by applying this framework to various forms of cyberostracism, we propose distinct pathways for different types of exclusion. Indirect exclusion and cyber-ignoring represent less severe and more ambiguous forms of ostracism that may not sufficiently deplete coping resources. When individuals encounter these less explicit forms of ostracism in one corner of the digital world (e.g., being ignored on Instagram), they cannot simply leave the online environment without sacrificing access to most of their social network. Instead, they may immediately re-engage with alternative apps or groups on the same device that may be more responsive, seeking approval from more accepting online communities to alleviate the social pain caused by exclusion (DeWall et al., 2010; Dong & Saini, 2023). Supporting this compensatory pattern, Büttner and Lutz (2025) found that individuals increase their social media use specifically after online ostracism. However, because they examined online ostracism as a general construct without distinguishing among its forms, it remains unclear whether this pattern applies to all types of exclusion. The present study addresses this gap by examining how different forms of cyberostracism are uniquely associated with PSU. Consequently, we expect indirect exclusion to be positively associated with PSU **(H1a)**. Similarly, we expect cyber-ignoring to be positively associated with PSU **(H1b)**. In contrast, direct exclusion represents an unambiguous, high-threat experience. Previous findings indicate that direct exclusion poses a greater threat to fundamental needs than cyber-ignoring, making it more likely to lead to resource depletion and resignation over time (Lutz & Schneider, 2021). Consequently, individuals who experience frequent direct cyberostracism may withdraw from the smartphone rather than engage in active coping. Accordingly, we expect a non-significant association between direct exclusion and PSU **(H1c)**.

### 1.2 Perceived parental phubbing as a mediator

According to the TNTM, individuals who are directly ostracized and withdraw from the exclusionary environment gradually become hypersensitive toward social threats while they are looking for more accessible and safer environments. In this context, parents remain an important source of emotional support even during young adulthood, and individuals prioritize parents over friends when seeking support (Guassi Moreira et al., 2018). This is particularly relevant given that explicit online exclusion most often occurs among peers and dating partners (Felmlee & Faris, 2016; Wang et al., 2019), yet peers are often passive bystanders rather than reliable supporters (Gahagan et al., 2016). Moreover, family supervision has been shown to buffer against peer-related online adversity (Elboj-Saso et al., 2024; Zhao et al., 2013). Yet, various barriers may prevent them from providing the attention and connection that their children expect (Zhang et al., 2024).

Parental phubbing refers to a child's perception of being neglected by their parents during interpersonal interactions due to parents' excessive attention to mobile phones, which interrupts or diminishes parent–child communication (Pancani et al., 2021; Pang et al., 2025). Critically, when parents act as phubbers, their emotional availability reduces significantly from the child's perspective (Solecki, 2022; Zhang, Dong, et al., 2023), thereby eliminating what would otherwise be the most accessible source of emotional support for ostracized youth. Given the increasing recognition of its negative impacts on parent-child relationships (Liu et al., 2024; Wang, Mao, et al., 2022) and children's both externalizing and internalizing issues, a growing body of findings has focused on its role in developing PSU (e.g., Liu et al., 2025; Mi et al., 2023; Shutao et al., 2024; Zhang et al., 2021). Thus, aligning with previous evidence, we expect perceived parental phubbing to be positively associated with PSU **(H2)**. However, few studies have examined the role of the child's early vulnerability such as exposure to different forms of online ostracism in creating such a harmful relationship. The Rejection Sensitivity Model (Downey & Feldman, 1996) provides a complementary framework that, when integrated with the TNTM, explains the link between cyberostracism and perceived parental phubbing. The synergy between the two models is as follows: the TNTM accounts for why individuals seek alternative offline relationships after experiencing direct ostracism. The Rejection Sensitivity Model, in turn, explains how these individuals come to interpret ordinary parental smartphone use as neglectful, because repeated exclusion heightens their anxious expectation of rejection, leading them to perceive ambiguous cues as signs of phubbing (Shi et al., 2025).

Consequently, when direct cyberostracism is followed by perceived parental phubbing, individuals appraise that they have been rejected in both online and offline contexts. Parents, their primary offline support, have failed them, leaving no equally accessible or trustworthy offline alternative. According to the TNTM, prolonged exposure to both online and offline ostracism not only hinders individuals from actively reacting to fortify their threatened needs but also leads them to a maladaptive response characterized by helplessness and alienation. In the digital context, we propose that PSU may manifest as one such maladaptive response. Rather than representing active, goal-directed coping, PSU may reflect a passive and compulsive engagement with the device, characterized by excessive scrolling, driven primarily by the constant accessibility of the smartphone. Supporting this, Marinucci et al. (2022) demonstrated that online social connections effectively substitute for offline interactions when face-to-face contact is not possible. By examining PSU as a potential passive response in the resignation stage, our study aims to develop this model by exploring how individuals may respond to this difficult situation, given that the application of the model to digital environments remains underexplored (Riva et al., 2026). It is important to distinguish this indirect pathway from the direct association between exclusion and PSU. While direct exclusion alone may not be sufficient to explain PSU (as individuals disengage from the online environment), the experience of being rejected by both online sources and parents creates a state of profound helplessness, making passive and compulsive smartphone use a likely maladaptive response. Therefore, we hypothesize that perceived parental phubbing mediates the relationship between direct exclusion and PSU **(H3a)**.

In contrast, for indirect exclusion and cyber-ignoring, individuals are less likely to disengage from their online social environment or develop heightened rejection sensitivity toward parents. When needs are threatened to a lesser extent, individuals are more likely to adopt approach behaviors, including connecting with others on social media to restore their threatened needs (Büttner et al., 2025). Consistent with this, a diary study by Lutz (2023) revealed that experiencing ostracism through messaging platforms strengthened users' motivation to return to those same platforms the next day, indicating a coping response oriented toward seeking reconnection online instead of pulling away from the digital environment. Thus, in this case, remaining in the same environment where the exclusion occurred and persistently trying harder to reestablish optimal levels of the threatened needs may serve as a better option than using another context, such as offline relationships, to fortify those needs. Therefore, perceived parental phubbing is not expected to mediate the relationship between indirect exclusion **(H3b)** or cyber-ignoring **(H3c)** and PSU.

### 1.3 Current study

In summary, the present study aimed to examine the differential associations between three forms of cyberostracism and PSU, as well as the mediating role of perceived parental phubbing in these relationships among university students. The rationale for choosing this population is that university students experience frequent social network changes that heighten their sensitivity to exclusion (Smith et al., 2017), and young adults, particularly college students, are among the most active and frequent users of social networking platforms (Tang & Duan, 2023). To our knowledge, although earlier studies have found that in-person ostracism was positively associated with PSU (Sun et al., 2023), it remains unclear whether similar patterns also apply to online forms of exclusion (Jung Kim et al., 2012). By focusing on different subtypes of cyberostracism and their unique associations with PSU, the present study addresses the previous research gap which has largely treated cyber-ostracism as a unitary construct (Xing & Kuo, 2024), yet empirical evidence shows that different forms of online exclusion have distinct psychological effects (Lutz & Schneider, 2021). Accordingly, this study seeks to extend current understanding of social-interpersonal stressors involved in the development and maintenance of PSU and to highlight the role of family-related digital behaviors as a potential explanatory mechanism. Furthermore, the findings may provide a conceptual foundation for future research on social exclusion and technology-related problematic behaviors, as well as for the development of socially informed prevention and intervention strategies in digital contexts.

## 2 Method

### 2.1 Participants and procedure

Our sample included 428 Iranian university students (303 females, 125 males; M = 28.03, SD = 8.67). Participants were recruited via convenience sampling in 2025. Inclusion criteria were being a current university student in Iran and willing to participate voluntarily; exclusion criteria were lack of informed consent or incomplete responses. Data were collected using a self-report questionnaire available online (via Porsline) and in paper format. The survey link was shared through Telegram and WhatsApp groups for university students, ensuring only enrolled students had access. Participants first viewed a summary of the study's goals and their rights, provided informed consent via a checkbox, and completed the questionnaire in approximately 15 min.

### 2.2 Measures

#### 2.2.1 Cyberostracism scale (CS)

Participants' perceptions of online social exclusion were measured using the 14-item Cyberostracism Scale (CS; Hatun & Demirci, 2022), comprising three dimensions: Cyber Direct Exclusion, Cyber Indirect Exclusion, and Cyber Ignoring. Items were rated on a 5-point Likert scale (1 = Never to 5 = Always) reflecting experiences over the past year on platforms such as Facebook, Instagram, Twitter, and WhatsApp. Sample items include: “They block me on social media” (Direct), “They share important messages in a group where I was not involved” (Indirect), and “They do not read my posts” (Ignoring). The three-factor structure was confirmed via CFA in the Persian version by the following indices: χ^2^ (73, *N* = 428) = 258.42, *p* < 0.001, CFI = 0.94, RMSEA = 0.077 (90% CI = [0.067, 0.087]), RMR = 0.031. Additionally, the scale exhibited acceptable internal consistency in the current sample (α = 0.92 overall; 0.89 Ignoring, 0.76 Direct, 0.81 Indirect).

#### 2.2.2 Smartphone application-based addiction scale (SABAS)

Problematic smartphone use (PSU) was assessed using the 6-item SABAS (Csibi et al., 2018), rated on a 6-point Likert scale (1 = Strongly Disagree to 6 = Strongly Agree), with higher scores indicating greater PSU. Sample item: “My smartphone is the most important thing in my life.” Lin et al. (2019) validated the Persian version of the scale in a sample of Iranian adolescents, finding it to be unidimensional and demonstrating good internal consistency (α = 0.86). The scale also showed satisfactory internal consistency in our sample (α = 0.85).

#### 2.2.3 Perceived parental phubbing scale (PPPS)

Parental phubbing was measured using the 9-item Perceived Parental Phubbing Scale (PPPS), which has been validated among Iranian university students (Mohammadi & Ghanbari, 2026). Respondents indicated how frequently they experienced parental phubbing behaviors on a 5-point Likert scale, ranging from 1 (Never) to 5 (All the time), with higher scores reflecting greater perceived parental phubbing. An example item from the scale is: “My parents glance at their phones while talking to me.” Internal consistency in our sample was high (α = 0.90).

### 2.3 Statistical analysis

All analyses were conducted using SPSS 26 and AMOS 26. Descriptive statistics summarized sample characteristics and variable distributions. Pearson correlations examined bivariate associations, and a one-way ANOVA tested differences across age categories. Assumptions of normality, linearity, and multicollinearity were adequately met.

The hypothesized mediation model was tested using SEM in AMOS, specifying Perceived Parental Phubbing as a mediator between cyberostracism and PSU. Indirect effects were evaluated via bias-corrected bootstrapping with 2000 resamples to obtain robust estimates and 95% confidence intervals. Model fit was assessed using standard indices (e.g., RMSEA, CFI).

## 3 Results

### 3.1 Preliminary analyses

Table 1 presents the descriptive statistics and bivariate correlations among the study variables. All cyberostracism subdimensions, perceived parental phubbing, and PSU were positively intercorrelated, providing preliminary support for the hypothesized relationships.

**Table 1:** Means, standard deviations, and Pearson correlations for scale scores.

Variable	M (SD)	1	2	3	4	5
1. CDE	6.32 (2.59)	1				
2. CIE	5.59 (2.52)	0.61⁎⁎	1			
3. CI	9.25 (4.06)	0.70⁎⁎	0.73⁎⁎	1		
4. PPPS	17.13 (6.88)	0.41⁎⁎	0.40⁎⁎	0.40⁎⁎	1	
5. PSU	17.63 (6.82)	0.27⁎⁎	0.30⁎⁎	0.24⁎⁎	0.29⁎⁎	1

Note. M = Mean; SD = Standard deviation; CDE = Cyber direct excluded; CIE = Cyber indirect excluded; CI = Cyber ignored; PPPS = Perceived parental phubbing; PSU = Problematic smartphone use.

⁎⁎ Correlation is significant at *p* < 0.01 (two-tailed).

One-way ANOVAs examined age differences. Cyberostracism subdimensions and parental phubbing did not differ across age groups, but a significant age difference was found for PSU. Post hoc Bonferroni tests indicated that participants aged 18–25 reported higher PSU than those aged 25–40 and 40–65, with no difference between the latter two groups (see Table 2).

**Table 2:** Age differences for the scale scores using analyses of variance.

Variable	**18–25**: M(SD)	**25–40**: M(SD)	**40–65**:M(SD)	F(2,425)	p	η2 p
1. CDE	6.43 (2.81)	6.18 (2.43)	6.23 (1.83)	0.48	0.621	0.002
2. CIE	5.79 (2.73)	5.32 (2.25)	5.54 (2.16)	1.65	0.194	0.008
3. CI	9.46 (4.42)	8.79 (3.62)	9.95 (3.45)	1.93	0.147	0.009
4. PPPS	17.57 (7.23)	16.68 (6.63)	16.36 (5.71)	1.07	0.344	0.005
5. PSU	18.83 (7.09)	16.34 (6.29)	15.95 (6.02)	7.86	<0.001	0.036

Note. CDE = Cyber Direct Excluded; CIE = Cyber Indirect Excluded; CI = Cyber Ignored; PPPS = Perceived Parental Phubbing; PSU = Problematic Smartphone Use.

### 3.2 CFA results

The results of the confirmatory factor analysis indicated that the measurement model demonstrated an acceptable fit to the data. As shown in Table 3, most goodness-of-fit indices met the recommended criteria. Specifically, the χ^2^/df and RMSEA values were within acceptable ranges, and the comparative fit indices (CFI and IFI) exceeded the recommended threshold of 0.90. Although GFI and RFI were slightly below the conventional cutoff, they were close to the recommended values. Taken together, these findings suggest that the measurement model provided a satisfactory representation of the observed data.

**Table 3:** Confirmatory factor analysis (CFA) and fit indices.

Goodness of Fit index	Model Fit	Recommended Value	Decision
RMSEA (CI 90%)	0.06 (0.055–0.065)	*≤ 0.08*	Good Fit
_sb_X^2^	934.79	*–*	–
CMIN/DF	2.55	*≤ 3*	Good Fit
CFI	0.91	*≥ 0.90*	Good Fit
IFI	0.91	*≥ 0.90*	Good Fit
RFI	0.85	*≥ 0.90*	Close
AGFI	0.84	*≥ 0.80*	Good Fit
GFI	0.87	*≥ 0.90*	Close
RMR	0.06	*≤ 0.08*	Good Fit

Note. RMSEA = Root Mean Square Error of Approximation; CFI = Comparative Fit Index; IFI = Incremental Fit Index; RFI = Relative Fit Index; AGFI = Adjusted Goodness of Fit Index; GFI = Goodness of Fit Index; RMR = Root Mean Square Residual.

To address concerns regarding common method bias due to cross-sectional self-report data, we conducted an unmeasured latent method construct (ULMC) analysis. A common latent factor (CLF) was added to the measurement model, with all items loading onto both their respective constructs and the CLF. The model with the CLF showed improved fit (χ^2^/df = 2.25, CFI = 0.93, RMSEA = 0.054) compared to the measurement model without the CLF (χ^2^/df = 2.55, CFI = 0.91, RMSEA = 0.06). Specifically, the CFI increased by only 0.02 (from 0.91 to 0.93), which is well below thresholds typically indicating substantial common method bias. These results suggest that common method bias does not substantially threaten the validity of our findings.

### 3.3 Mediation analysis

**Fig. 1:**
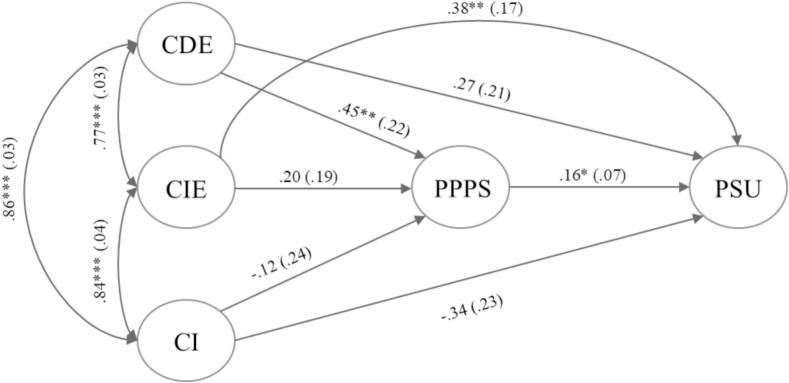
SEM model with standardized path coefficients (and standard errors in parentheses). Note. CDE = Cyber Direct Excluded; CIE = Cyber Indirect Excluded; CI = Cyber Ignored; PPPS = Perceived Parental Phubbing; PSU = Problematic Smartphone Use. Double-headed arrows represent covariances between exogenous variables. For simplicity of presentation, the latent variable factor loadings are not displayed; nonetheless, the authors can supply them upon request. *p < .05. ** *p* < .01. *** *p* < .001.

Bootstrapping analyses (with 2000 resamples) were conducted to examine whether perceived parental phubbing mediated the relationship between cyber-ostracism subdimensions and PSU (see Fig. 1 and Table 4). For the cyber-direct exclusion subdimension, the results supported a full mediation (indirect-only) pattern, indicating that the association between cyber-direct exclusion and PSU was fully mediated by perceived parental phubbing. In contrast, for the cyber-indirect exclusion subdimension, the findings revealed a direct-only association, as the indirect pathway via perceived parental phubbing was not significant. Finally, for the cyber-ignoring subdimension, neither the direct nor the indirect pathways were significant, suggesting no evidence of an association with PSU through perceived parental phubbing.

**Table 4:** Mediating role of perceived parental phubbing in the association between cyber-ostracism subdimensions and problematic smartphone use.

**Subdimension**	**Effect**	**β**	**CI 95%**	***p*-value**
Cyber direct excluded	Total	0.344	[0.030,0 .715]	**0.036**
	Direct	0.273	[-0.081, 0.625]	0.138
	Indirect	0.071	[0.011, 0.208]	**0.019**
Cyber indirect excluded	Total	0.412	[0.086, 0.763]	**0.017**
	Direct	0.381	[0.066, 0.733]	**0.019**
	Indirect	0.031	[-0.016, 0.115]	0.176
Cyber ignored	Total	-0.356	[-0.88, 0.003]	0.052
	Direct	-0.337	[-0.843, 0.029]	0.070
	Indirect	-0.019	[-.138, 0.034]	0.344

*Note.* Significant coefficients (*p* < .05) are bolded.

## 4 Discussion

The present findings reveal a nuanced pattern linking different forms of cyberostracism with PSU and clarify how perceived parental phubbing contributes to this process. Grounded in established models of social–interpersonal functioning, the results show that social media ostracism operates as a meaningful risk factor beyond digital interactions (Skogen et al., 2023). Rather than eliciting only momentary emotional discomfort, cyber-based exclusion appears to shape how individuals interpret offline relational cues and how they regulate their engagement with smartphones.

Consistent with H1a, cyber-indirect exclusion showed significant positive associations with PSU. This finding is conceptually consistent with the TNTM, which posits that ostracism threatens four fundamental needs, motivating individuals to employ compensatory coping strategies. In the context of smartphone use, social media platforms serve as readily accessible tools that provide immediate, though temporary, restoration of social connection and validation (Ding et al., 2022; Schneider et al., 2017; Xing & Kuo, 2024). Consequently, compensatory reliance on these platforms may become a predictable strategy for redefining self-worth. This pattern of compensatory reconnection is further supported by recent large-scale data from social media platforms where users who received fewer Likes than usual on their posts subsequently posted again more quickly, reflecting acknowledgment-seeking behavior (Kenntemich et al., 2026). Over time, this compensatory reliance may correspond to PSU, where the smartphone plays a central role in alleviating exclusion-related distress. Such dynamics are consistent with findings showing that interdependent self-construal can facilitate rebounding from ostracism (Ren et al., 2013). For example, individuals may make real-life efforts to reach out to others and regain social inclusion through prosocial behavior (Choy et al., 2021; Williams & Nida, 2022), which increasingly occurs via digital platforms and may evolve into maladaptive patterns of smartphone engagement.

Nonetheless, not all evidence supports prosocial coping after exclusion (Lutz et al., 2024; Xiao & Wang, 2025). For example, Kip et al. (2025) found that participants exposed to experimentally induced ostracism in a Cyberball task primarily withdrew rather than attempted to reconnect. A recent meta-analysis likewise reported an overall weak negative association between social exclusion and prosocial behavior, suggesting that prosocial coping is not the dominant pattern across contexts (Lin et al., 2025). Taken together, these findings are consistent with the non-significant association observed between cyber-direct exclusion and PSU in the present study, supporting H1c. Specifically, when youth are directly excluded in cyberspace, their willingness to engage in cooperation and reintegration appears to be weakened (Twenge et al., 2007). Experimental research further indicates that when cyberostracism is explicitly imposed by others, individuals predominantly engage in withdrawal rather than prosocial coping (Lutz et al., 2024).

Interestingly, contrary to H1b, cyber-ignoring was not significantly associated with PSU. We interpret this finding as suggesting that cyber-ignoring may be experienced as less personally threatening and less indicative of intentional exclusion, particularly compared to indirect exclusion which involves active omission from social interactions. Consequently, cyber-ignoring may not correspond to the same level of compensatory reconnection motivation. This interpretation aligns with previous research showing that ambiguous neglect evokes weaker psychological responses than explicit rejection (Lutz & Schneider, 2021). Overall, these results underscore the importance of distinguishing among subdimensions of cyberostracism rather than treating it as a unitary construct. Future research should examine psychological and behavioral responses to different forms of cyberostracism across diverse contexts.

Another major contribution concerns perceived parental phubbing. Consistent with theoretical expectations and prior research, perceived parental phubbing had a significant positive relationship with PSU (H2), indicating that relational neglect during parent–child interactions represents an interpersonal risk factor for excessive smartphone use among young adults. While this association has been documented in adolescents (Mi et al., 2023; Shutao et al., 2024), the present findings extend it to older age groups and different cultural contexts. From this perspective, individuals' subjective experiences of parental emotional disengagement and unavailability may relate to higher vulnerability to maladaptive smartphone use as a compensatory strategy. In low-quality family relational climates, smartphones may serve as an easily accessible means of distancing from interpersonal discomfort and regulating negative emotions arising from being phubbed (Pang et al., 2025; Zhang & Wang, 2025).

Moreover, beyond this direct association with PSU, perceived parental phubbing was significantly higher among individuals who reported direct cyberostracism, suggesting a distinct interpersonal pathway through which explicit online exclusion may be related to offline family perceptions. Guided by the Rejection Sensitivity Model, heightened sensitivity following direct cyberostracism may correspond to individuals interpreting parental smartphone use as a more salient signal of rejection, particularly within the parent–child relationship. Building on this framework, the results further indicated that perceived parental phubbing emerged as a significant mediator only in the pathway between cyber-direct exclusion and PSU, supporting H3a. This significant mediation is consistent with a sequential process, whereby explicit and unambiguous exclusion in cyberspace may be associated with disengagement and withdrawal from the context in which exclusion occurred rather than immediate social reconnection (Chen et al., 2023), in line with experimental evidence indicating that direct social exclusion is associated with reduced perceived desirability of belongingness goals (Rühs et al., 2022). As outlined in our theoretical framework, such prolonged exposure to ostracism takes individuals to the resignation stage, which is accompanied by a range of maladaptive responses that reflect the individual's deteriorated psychological state and helplessness resulting from perceived chronic ostracism in both online and offline environments. In this context, turning to phones passively and compulsively may represent one such response and may be associated with PSU. This interpretation is consistent with robust evidence showing an association between PSU and depressive symptoms, suggesting that these maladaptive reactions are associated with chronic social pain rather than active coping efforts (Augner et al., 2023; Tang et al., 2024). Future research could also examine other digital behavioral manifestations of the resignation stage following chronic ostracism, such as cyberbullying or cyberloafing, thereby extending our understanding of digital responses beyond PSU. Thus, parental phubbing represents a critical interpersonal factor in the breakdown of offline support and subsequent passive smartphone engagement.

In contrast, for indirect exclusion and cyber-ignoring, perceived parental phubbing did not mediate the relationship with PSU, consistent with H3b and H3c. Conversely, when cyberostracism is more subtle and less threatening (cyber-ignoring), or when it is primarily associated with social reconnection through increased smartphone engagement to satisfy threatened needs (cyber-indirect exclusion), parental phubbing plays no significant intervening role. Notably, although all three cyberostracism subtypes correlated similarly with perceived parental phubbing (Table 1), these bivariate associations reflect both unique and shared variance among the subtypes. When this shared variance was taken into account in our main model, however, only direct exclusion remained uniquely linked to parental phubbing and, through it, to PSU. This suggests that the associations of indirect exclusion and cyber-ignoring with parental phubbing are largely explained by their overlap with direct exclusion, a pattern consistent with our theoretical distinction.

Taken together, these findings underscore the complex interplay between online and offline social dynamics in shaping young adults' technology-related behaviors. These findings reveal a specific sequential pattern for the direct form of cyberostracism under which individuals become ‘doubly excluded’: higher levels of direct cyber exclusion are associated with higher levels of perceived parental phubbing, which in turn are associated with higher levels of PSU. By jointly examining cyberostracism and perceived parental phubbing, the present study suggests that PSU is better understood when considering broader interpersonal contexts, particularly family-related digital habits. The observed mediation effects are consistent with socially grounded models of PSU and an integrated view of social exclusion, where online and offline experiences are interrelated. Practically, reducing PSU may benefit from encouraging parents to adopt mindful smartphone habits and maintain emotional availability, thereby enhancing family communication. Preventive efforts should also aim to create inclusive digital contexts that reduce passive and compulsive smartphone use as a response to social exclusion.

## 5 Limitations and future directions

Despite its strengths, this study has several limitations. First, the cross-sectional design and reliance on self-report data inherently limit causal inference and introduce potential biases, including recall bias, interpretation bias (especially given the subjective nature of exclusion experiences), and common method bias (CMB). Although our ULMC analysis suggested that CMB was not a serious threat, some degree of CMB may still exist due to the nature of cross-sectional self-report designs. Future research should consider longitudinal or multi-method approaches to address these limitations. Second, the study focused on perceived parental phubbing rather than parents' actual smartphone use; future research could incorporate parent-reported or observational data to better capture family digital interactions. Third, cultural factors in the Iranian context may influence interpretations of parental behavior and coping responses to online exclusion, warranting cross-cultural studies Fourth, while our theoretical framework draws on the TNTM's four fundamental needs (belonging, self-esteem, control, and meaningful existence), we did not directly measure these psychological needs. Future research should incorporate measures of these needs to compare their explanatory contribution with the interpersonal pathway examined here. Fifth, we did not measure negative emotions, which have been shown to be associated with both online ostracism (Bevan et al., 2012) and PSU (Zhong et al., 2025). Future research should include negative emotions as a covariate to rule out potential confounding effects. Future research could also examine additional regulatory mechanisms aligned with this framework, such as emotion regulation or compulsive reassurance seeking. Sixth, while we focused on parental phubbing, we acknowledge that other close relationships such as siblings, close friends, or romantic partners may serve similar functions. Expanding interpersonal contexts to include peer or romantic partner phubbing may further clarify how digital exclusion across relationships contributes to PSU.

## 6 Conclusion

In summary, this study advances understanding of PSU by showing that cyberostracism operates through distinct psychological pathways. Indirect online exclusion was correlated with PSU by motivating compensatory smartphone engagement to restore threatened needs, while direct cyberostracism showed a relationship with PSU mainly when it corresponded to altered perceptions of offline relational availability, particularly in the parent–child context. Perceived parental phubbing functioned as an explanatory mechanism in the relationship between online direct exclusion and PSU, highlighting the need to integrate cyberostracism with family dynamics in theoretical models. Interventions may benefit from addressing both digital exclusion experiences and parents' smartphone practices, emphasizing emotional availability during parent–child interactions. These results also underscore the importance of distinguishing among subdimensions of cyberostracism, rather than treating online exclusion as a single construct, when examining technology-related maladaptive behaviors.

## CRediT authorship contribution statement

**Mehran Mohammadi:** Writing – review & editing, Writing – original draft, Project administration, Methodology, Investigation, Formal analysis, Data curation, Conceptualization. **Nikzad Ghanbari:** Writing – review & editing, Validation, Supervision, Methodology, Investigation, Formal analysis.

## Ethics approval

In accordance with the Declaration of Helsinki and applicable national guidelines, this research followed ethical principles for human participant research. We obtained informed consent from all participants after ensuring they understood the study's objectives, confidentiality protocols, and their rights. Participation was voluntary and anonymous, and participants were informed of their right to decline questions or withdraw at any time without penalty. Participant privacy was strictly maintained. This procedure was approved by the Research Ethics Committee of Shahid Beheshti University.

## Declaration of generative AI and AI-assisted technologies in the writing process

ChatGPT was used only to improve language clarity and fluency. Scientific content, data analysis, and conclusions were fully generated and verified by the authors, who take full responsibility for the final manuscript.

## Declaration of competing interest

The authors declare that they have no known competing financial interests or personal relationships that could have appeared to influence the work reported in this paper.

## Data Availability

Data will be made available on request.
